# Peer Review of “Human Brucellosis in Iraq: Spatiotemporal Data Analysis From 2007-2018”

**DOI:** 10.2196/60394

**Published:** 2024-07-03

**Authors:** Anonymous

**Keywords:** human brucellosis, livestock, clustering, spatial, temporal, Iraq

*This is the peer-review report for “Human Brucellosis in Iraq: Spatiotemporal Data Analysis From 2007-2018.”*

## Round 1 Review

### General Comments

This paper [1] talks about human brucellosis in Iraq and brings an interesting spatiotemporal analysis of the human cases in the country. The paper will contribute to the understanding of human brucellosis in Iraq and can be one more example of the use of spatiotemporal analysis for the control of the disease. However, some changes need to be made to clarify some information in the paper.

### Specific Comments

#### Major Comments

##### Introduction

1. References are missing in the second phrase of the second paragraph.

2. In the third phrase, “(dogs)” is not necessary.

3. The breeding season of sheep and goats is not in spring. So, the phrase “However, it usually coincides with the livestock breeding season, spring” should be changed, as well as “Human exposure to livestock or their contaminated products will occur during spring.” The second part of this phrase is true but not absolute, since animal products can travel to other places; so for clarity, I think this phrase should be reformulated.

4. Please, develop this paragraph further: “A study from northern Iraq showed that the prevalence of brucellosis in livestock varied from 1% to 70%, depending on the species and diagnostic methods. 4 Veterinary vaccination program started in 2007. However, its implementation was negatively affected by insecurities in the region.4.” It would be very interesting to know more about the testing and the insecurities of the population regarding vaccination.

5. Please connect more the idea of the first phrase of the fourth paragraph with the following ideas.

6. Please define “MOH’s” in the last paragraph.

##### Methods

7. Please make it clear throughout that the data are about humans and not animals.

8. In the data description, why does the data come from different sources and with different types of organization and grouping? Can you make it clearer?

9. What is HB? Please define it before using the abbreviation.

10. What is period one?

11. What does “the low or high attribute zone” mean?

12. Please explain the *P* values assumed for the “Getis-Ord Gi* statistic” and what exactly this statistic is identifying.

13. In the *Abstract*, the statistical analysis is explained differently from the *Methods*. Please make them similar.

##### Discussion

14. Have you tested the trend? If so, please clarify the methods and results, but if not, please reformulate the phrase where the word is being used to describe the occurrence of your data.

15. Can you define what were the “ISIL events”?

16. “The number of females has been constantly higher than males.” You are talking about humans, please used woman and man and clarify, in the *Methods* section, whether this classification was self-made or not.

17. “housekeeping and farming activities. 5 (11, 12,13).” I did not understand the different configuration of the references in here.

18. “However, this age category was very broad and could have been classified into two to three age groups to detect the most commonly affected age group. (14-17).” Why was this change not made? Again, the references are in a strange configuration.

19. “The infected animals must be eradicated by slaughtering and burning because there is no curable medical therapy for animal brucellosis.” I did not understand this phrase. Please remove the word eradication from here and explain better why you are saying that the animals should be burned. I do understand that positive animals should be slaughter and their carcasses should be disposed of in the right way, but I have not heard of burning before.

20. “On the other hand, humans may consume this infected milk unpasteurized, resulting in infection and areas endemic with brucellosis to animals and humans.” Change “to” for “in.”

21. There is an “18” in paragraph 5 that could be a reference. I did not understand the phrases that followed the 18.

22. The paragraph “Transboundary transfer of animal brucellosis in the region from the neighboring countries such as Iran, Turkey, and Syria were provoked by war and political instability, lack of immunization and animal quarantine, frequent trading, low awareness and poor knowledge of HB prevention and control, residents with poor sanitary conditions easily exposed to Brucella contaminated food and water sources.” is disconnected from the rest of the text; I did not understand what exactly this is about.

23. The first and second periods of the study are not clear for me.

##### Conclusion

24. The last part of the conclusion would be better in the *Discussion* section, such as “Preventive measures such as health education activities should be performed in high-risk areas. Adopting the Quarantine-Slaughter-Immunization strategy and One Health Approach is crucial in controlling the disease. This can be achieved through multisectoral coordination and coordination with neighboring countries in the control programs.”

## Round 2 Review

### General Comments

This paper brings important information and analysis on human brucellosis in Iraq. To improve the paper’s understanding, I suggest an English review of the paper to improve the writing of the paper.

### Specific Comments

#### Major Comments

1. *Abstract*, section *Methods*: “The trend of cases by sex and age group were displayed from 2007‐2018 were displayed.” Please delete the last “were displayed.”

2. “The seasonal distribution of the cases from 2007 to 2012 was graphed.” Substitute “was” for “were.”

3. *Introduction*: The paragraph on the percentages of occurrence of brucellosis only present the values but does not make a value judgment or interpret what these values mean, why are they important, and so on. Please, reformulate again.

4. *Discussion*: Second paragraph: Make it clear that the number of woman you are talking about is the number of woman positive for brucellosis among the analyzed years.

5. *Conclusion*: Substitute HB for human brucellosis.

## References

[1] Mustafa AH, Khaleel HA, Lami F (2024). Human brucellosis in Iraq: spatiotemporal data analysis from 2007-2018. JMIRx Med.

